# Management of left traumatic diaphragmatic hernia complicated by traumatic Stanford type B aortic dissection

**DOI:** 10.1186/s44215-023-00104-8

**Published:** 2023-09-26

**Authors:** Yuta Matsubayashi, Yusuke Takanashi, Keigo Sekihara, Takamitsu Hayakawa, Kiyomichi Mizuno, Akikazu Kawase, Masanori Sato, Norihiko Shiiya, Kazuhito Funai

**Affiliations:** First Department of Surgery, Hamamatsu University School of Medicine, 1-20-1 Handayama, Higashi-Ku, Hamamatsu, Shizuoka 431-3192 Japan

**Keywords:** Traumatic diaphragmatic hernia, Aortic dissection, Elective surgery

## Abstract

**Background:**

Traumatic diaphragmatic hernias are frequently associated with multiple organ injuries caused by high-energy trauma. Herein, we report a case of left traumatic diaphragmatic hernia complicated by traumatic Stanford type B aortic dissection, in which we considered surgical strategies for the timing and approach of diaphragmatic hernia repair.

**Case presentation:**

A 65-year-old man was transported to our hospital following a traffic accident. He was diagnosed with left traumatic diaphragmatic hernia, traumatic Stanford type B aortic dissection, multiple fractures of the left ribs, hemothorax, and pulmonary contusion. Because acute surgery for hernia repair might exacerbate aortic dissection, we initiated conservative treatment for aortic dissection. Respiratory status and ischemia of the herniated organs were monitored carefully. On the day 6, when the aortic dissection was considered stable, we performed diaphragmatic hernia repair. A large surgical field secured by thoracolaparotomy enabled safe surgical techniques for visualization of the aortic wall. Postoperatively, there was no diaphragmatic hernia recurrence, and the aortic dissection remained stable with conservative treatment.

**Conclusions:**

In traumatic diaphragmatic hernia complicated by traumatic Stanford type B aortic dissection, elective surgery via the trans-thoracoabdominal approach may be safe after stabilization of aortic dissection, provided the respiratory condition can be kept stable.

## Background

Traumatic diaphragmatic hernia is associated with blunt thoracic trauma [1]. It is one of the most common injuries resulting from multiple organ damage caused by high-energy trauma, such as traffic accidents. Common treatment strategies for traumatic diaphragmatic hernia complicated by traumatic aortic injury have not yet been reported. In particular, timing and approach of surgical intervention need to be discussed. Herein, we report a case of left traumatic diaphragmatic hernia associated with traumatic Stanford type B aortic dissection, in which surgical strategies for the timing and approach should be discussed.

## Case presentation

**Fig. 1:**
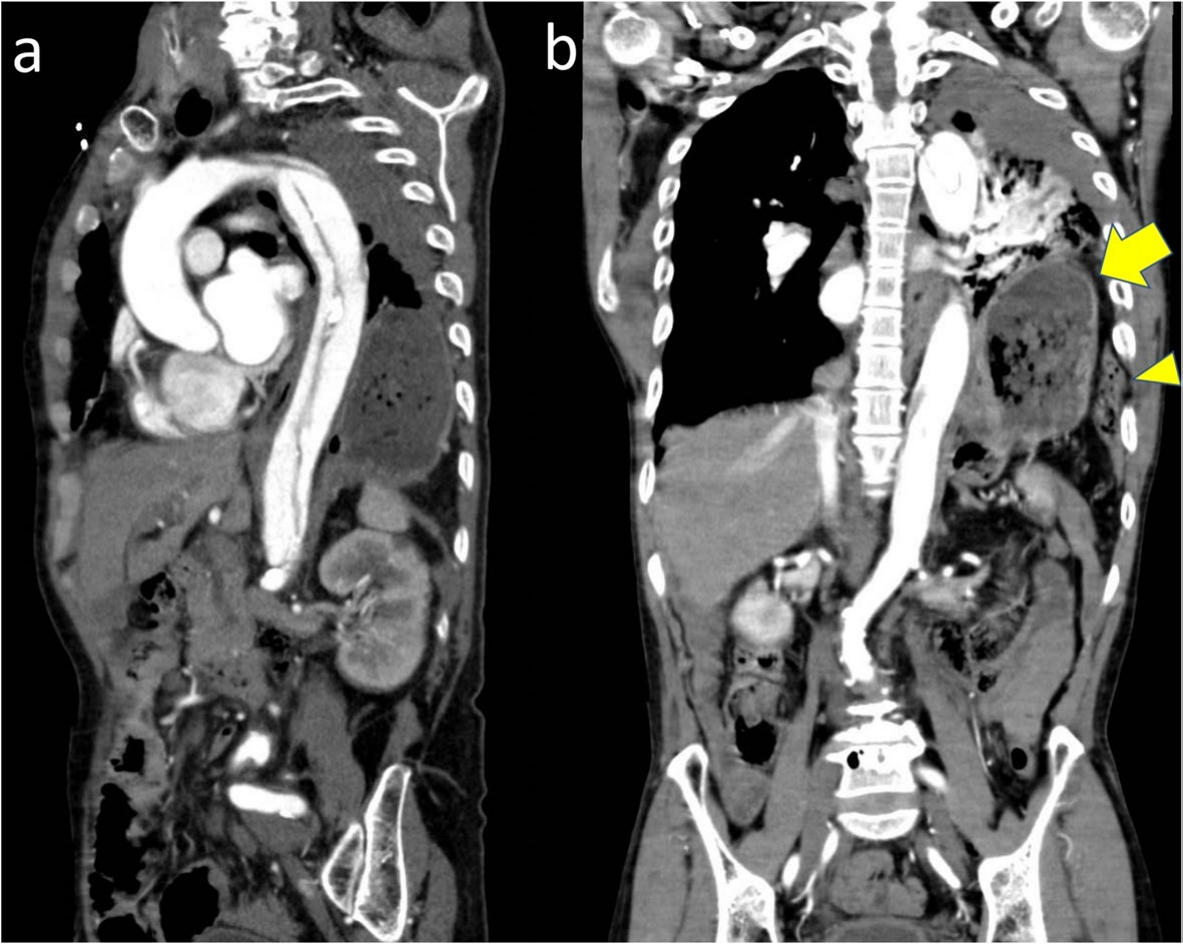
Contrast-enhanced computed tomography images at hospital arrival. Computed tomography showing aortic dissection from the level of the left subclavian artery to the celiac artery (**a**). In the coronal section, diaphragmatic rupture and herniation of the stomach (arrow), descending colon (arrow head) into the left thoracic cavity, and hemothorax are observed (**b**)

A 65-year-old man was transported to an emergency department of a previous hospital, because of a car accident. Contrast-enhanced computed tomography (CT) of the chest showed traumatic Stanford type B aortic dissection from the level of the left subclavian artery to the celiac artery (Fig. 1a), multiple fractures of the left ribs, left hemothorax, and left pulmonary contusion. He was referred to our hospital for treatment of the aortic dissection. Chest CT scan also demonstrated herniation of the stomach, descending colon, and left lobe of the liver into the left thoracic cavity (Fig. 1b). No obvious injury nor ischemia was seen in the herniated organs. The patient was hypoxemic (PaO_2_ 198.9 mmHg; PaCO_2_ 36.0 mmHg [12 L oxygen reservoir face mask]), which showed improvement by prompt initiation of mechanical ventilation. Laboratory data showed anemia and mild liver dysfunction: hemoglobin, 10.3 g/dL; total bilirubin, 0.4 mg/dL; aspartate transaminase, 152 U/L; and alanine transaminase, 43 U/L.

Although surgical treatment of diaphragmatic hernia is necessary to improve the respiratory condition and prevent incarceration of the abdominal organs, emergent operation during the acute phase might aggravate aortic dissection. After multi-disciplinary team discussions with thoracic, cardiovascular, and gastroenterological surgeons, we decided to perform diaphragmatic hernia repair after aortic dissection stabilization. Thus, conservative treatment for aortic dissection was initiated under mechanical ventilation and gastric decompression; blood pressure was controlled to maintain systolic blood pressure at 90–120 mmHg. The quantification of serum lactate levels was performed at 8-h intervals to evaluate the incidence of ischemia of the abdominal organs.

The respiratory condition and diaphragmatic hernia did not worsen. Contrast-enhanced CT on the day 5 showed no enlargement of the dissected aorta, and the aortic dissection was considered stable. The quantification of serum lactate levels during the preoperative period ranged from 0.5 to 0.8 mmol/L, indicating an absence of any discernible signs of ischemia of the abdominal organ. Diaphragmatic hernia repair was performed on the day 6. Given the potential for vascular rupture, the operation was performed in a hybrid operating room to facilitate the prompt initiation of thoracic endovascular aortic repair (TEVAR) in the event of such an occurrence. In order to visualize the descending aorta and avoid injury, a trans-thoracoabdominal approach was chosen, which provides a sufficient operative field.

**Fig. 2:**
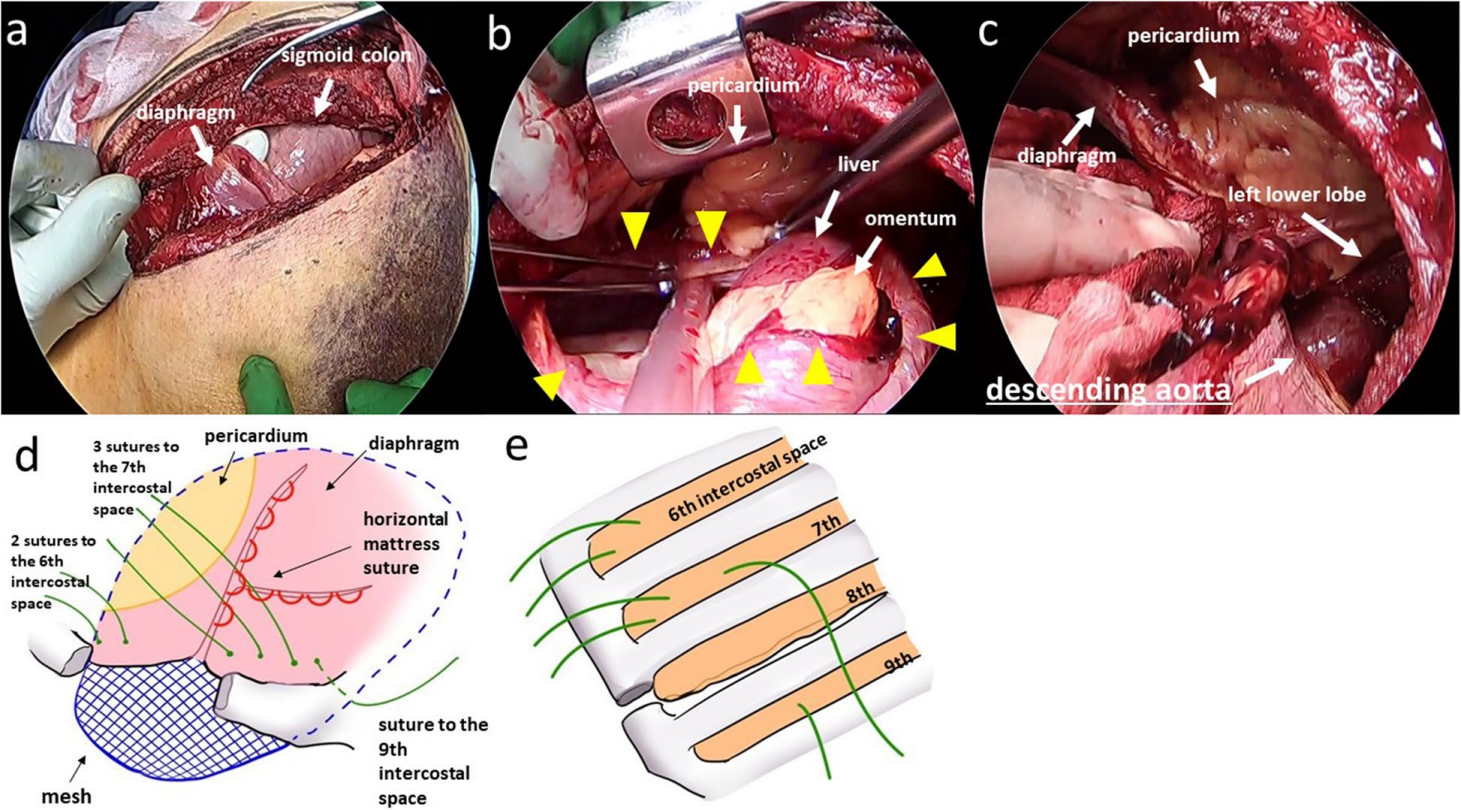
Intraoperative findings and the resulting schema. Surgical exploration showing diaphragmatic laceration and herniation of the sigmoid colon into the thoracic cavity. The finger passing from the abdominal cavity into the thoracic cavity (**a**). The forceps grasped the diaphragmatic stump attached to the pericardium. The diaphragm is widely ruptured (arrow heads), and the omentum and liver herniate into the thoracic cavity (**b**). The descending aorta, where aortic dissection had occurred, is visible in the thoracic cavity (**c**). The surgical schema depicts the repair of the diaphragm, as illustrated by the red curved lines, utilizing horizontal mattress suture fixation (**d**). The green suture lines indicate the fixation of the diaphragm and mesh to the sixth, seventh, and ninth intercostal spaces from the abdominal cavity (**e**)

In the right semi-lateral decubitus position, a 25-cm skin incision along the eighth intercostal space was placed from the posterior axillary line to the umbilicus. The eighth costal arch was divided to perform thoracolaparotomy. We smoothly repositioned the herniated abdominal organs into the abdominal cavity, as no adhesions, ischemic findings, or injury were observed (Fig. 2a). The diaphragmatic laceration extended from the left side of the pericardium to the ventral side of the costal arch (Fig. 2b, arrow heads). At the site of aortic dissection, there was a mixture of dark red thin-walled areas, and yellowish-white areas indicative of fibrosis (Fig. 2c). The large operative field made it apparent that the aortic wall had been stabilized with conservative treatment, and allowed for safe surgery. The diaphragmatic laceration was closed using a horizontal mattress suture with braided polyamide (Fig. 2d). A 15 × 10 cm mesh (TiLENE mesh, PFM Medical, Germany) was placed on the abdominal aspect of the diaphragm. The diaphragm was repaired along with the mesh by horizontal mattress suture fixation to the sixth, seventh, and nineth intercostal spaces from the abdominal cavity (Fig. 2e).

The patient was extubated on the first postoperative day, followed by removal of the thoracic drain. A minitracheostomy tube was inserted to alleviate sputum retention until the postoperative day 34. No recurrence of the diaphragmatic hernia was observed during the postoperative course. Aortic dissection was successfully treated conservatively, and the patient was discharged on the postoperative day 50.

## Discussion

The incidence of diaphragmatic hernia in patients with blunt trauma ranges between 0.8 and 1.6% [2]. Rib fractures, hemothorax, and intra-abdominal organ injuries are the most common traumatic injuries associated with diaphragmatic hernia, while thoracic aortic injuries account for only 1.0–7.7% of the cases [3, 4]. Moreover, traumatic aortic dissection is extremely rare among thoracic aortic injuries [5]. A traumatic diaphragmatic hernia requires surgical treatment, as soon as the diagnosis is made. However, because traumatic aortic dissection was associated, we needed to consider the timing of the surgical intervention and approach.

First, the timing of the surgical intervention is discussed. There was no significant difference in mortality between patients with traumatic diaphragmatic hernia who underwent early or elective surgery. Moreover, diaphragmatic hernia itself is an uncommon cause of death [3]. When a diaphragmatic hernia is a multiple-organ injury, the prognosis is determined by concomitant organ injury. Therefore, diaphragmatic hernia repair is not necessary in patients with a stable general condition in the acute phase of the disease.

Type B aortic dissection with no signs of malperfusion or disease progression was defined as “uncomplicated type” [6]. TEVAR is often used as a non-invasive surgical treatment for aortic dissection. However, TEVAR may cause serious complications, such as retrograde type A aortic dissection and spinal cord disorders [7]. In particular, in the acute phase of aortic dissection, application of TEVAR may cause retrograde type A aortic dissection, more likely due to fragility of the aortic wall. Acute type B aortic dissection has a low mortality rate of 13%, and conservative management for uncomplicated type B aortic dissection is associated with superior survival outcomes compared to surgical intervention [6]. Thus, conservative therapy is recommended in the acute phase of uncomplicated type B aortic dissection [8].

In this case, we considered the three strategies as follows: (i) immediate diaphragmatic hernia repair; (ii) immediate TEVAR followed by diaphragmatic hernia repair; and (iii) elective diaphragmatic hernia repair. Regarding immediate diaphragmatic hernia repair, hernia repair was considered to have a high risk of injury to the dissected aorta, owing to fragility of the aortic wall. Hernia repair was better performed after stabilization of the aortic dissection. As for immediate TEVAR, the risk of retrograde type A aortic dissection is high in the acute phase [9]. As the patient had uncomplicated type B aortic dissection, conservative treatment was appropriate in our case. We decided to perform elective diaphragmatic hernia repair for several reasons. The patient’s hemodynamic and respiratory conditions were stable under mechanical ventilation. Moreover, no injury or ischemia of herniated organs was observed. Therefore, we considered that hernia repair could be postponed until stabilization of the aortic dissection. Moreover, since traumatic aortic dissection is potentially more fatal than diaphragmatic hernia, conservative treatment for aortic dissection should be prioritized. As a result, the patient was safely treated using this strategy.

Finally, we discuss the surgical approach to diaphragmatic hernia repair. Generally, the following three approaches are chosen according to the site of the life-threatening wound, patient's hemodynamic stability, and surgeon’s preference and skill: trans-abdominal, trans-thoracic, and trans-thoracoabdominal approaches [10]. The trans-abdominal approach is recommended for exploration and repair of abdominal organs in acute diaphragmatic hernia. In contrast, the trans-thoracic approach is necessary in cases associated with intrathoracic injury, or to safely dissect adhesions between the abdominal organs and chest wall in cases of late diaphragmatic hernia [11]. The approach should be selected based on the patient’s general condition and presence and severity of complications. In our case, the aortic dissection was life-threatening. Therefore, the trans-thoracoabdominal approach was chosen to secure a large enough operative field to avoid injury to the descending aorta.

In a case of traumatic diaphragmatic hernia complicated by traumatic Stanford type B aortic dissection, elective surgery via the trans-thoracoabdominal approach after stabilization of aortic dissection should be considered, as long as the patient’s general condition can be kept stable.

## Data Availability

Not applicable.

## Abbreviations

CT: Computed tomography
TEVAR: Thoracic endovascular aortic repair
